# The Health and Economic Burdens of Lymphatic Filariasis Prior to Mass Drug Administration Programs

**DOI:** 10.1093/cid/ciz671

**Published:** 2019-07-25

**Authors:** Christopher G Mathew, Alison A Bettis, Brian K Chu, Mike English, Eric A Ottesen, Mark H Bradley, Hugo C Turner

**Affiliations:** 1 Centre for Tropical Medicine and Global Health, Nuffield Department of Medicine, University of Oxford, United Kingdom; 2 London Centre for Neglected Tropical Disease Research, Imperial College London, United Kingdom; 3 Department of Infectious Disease Epidemiology, School of Public Health, Faculty of Medicine, St Marys Campus, Imperial College London, United Kingdom; 4 Neglected Tropical Diseases Support Center, Task Force for Global Health, Decatur, Georgia; 5 Kenya Medical Research Institute, Wellcome Trust Research Programme, Nairobi; 6 Global Health Programs, GlaxoSmithKline, London, United Kingdom; 7 Oxford University Clinical Research Unit, Wellcome Africa Asia Programme, Ho Chi Minh City, Vietnam

**Keywords:** lymphatic filariasis, mass drug administration, NTD, GPELF, economic burden

## Abstract

**Background:**

The Global Programme to Eliminate Lymphatic Filariasis (GPELF) was launched in 2000 with the goal of eliminating lymphatic filariasis (LF) as a public health problem by 2020. Despite considerable progress, the current prevalence is around 60% of the 2000 figure, with the deadline looming a year away. Consequently, there is a continued need for investment in both the mass drug administration (MDA) and morbidity management programs, and this paper aims to demonstrate that need by estimating the health and economic burdens of LF prior to MDA programs starting in GPELF areas.

**Methods:**

A previously developed model was used to estimate the numbers of individuals infected and individuals with symptomatic disease, along with the attributable number of disability-adjusted life years (DALYs). The economic burden was calculated by quantifying the costs incurred by the health-care system in managing clinical cases, the patients’ out-of-pocket costs, and their productivity costs.

**Results:**

Prior to the MDA program, approximately 129 million people were infected with LF, of which 43 million had clinical disease, corresponding to a DALY burden of 5.25 million. The average annual economic burden per chronic case was US $115, the majority of which resulted from productivity costs. The total economic burden of LF was estimated at US $5.8 billion annually.

**Conclusions:**

These results demonstrate the magnitude of the LF burden and highlight the continued need to support the GPELF. Patients with clinical disease bore the majority of the economic burden, but will not benefit much from the current MDA program, which is aimed at reducing transmission. This assessment further highlights the need to scale up morbidity management programs.

## Teaser

Prior to mass drug administration, we estimate that lymphatic filariasis (LF) cost 5.25 million disability-adjusted life years and US $5.765 billion annually. The high burden of LF without control demonstrates the need for the Global Programme to Eliminate LF.

**(See the Editorial Commentary by Niessen and Taylor on pages 2568–9.)**

Lymphatic filariasis (LF) is a mosquito-borne neglected tropical disease (NTD). Clinical disease can manifest as severe fluid accumulation, generally in the limbs (lymphedema) or scrotal sac (hydrocele), or as episodes of acute adenolymphangitis (ADL) [1].

The Global Programme to Eliminate Lymphatic Filariasis (GPELF) was launched in 2000, with the goal of eliminating LF as a public health problem by 2020 [2, 3]. The GPELF proposed 2 strategies: interrupting transmission through mass drug administration (MDA); and morbidity management and disability prevention [2, 3]. Through long-term pledges of drug donations from pharmaceutical companies, MDA has been conducted at scale, providing over 7.1 billion treatments in endemic areas since 2000 [4]. By 2018, of the 72 endemic countries, 14 had been validated by the World Health Organization (WHO) to have eliminated LF, with 7 conducting post-MDA surveillance [5].

Since the global program is around 40% complete [4], there is a need for continued investment to ensure that global LF elimination is successfully achieved by 2030. The third WHO NTD report set domestic investment targets for NTD control to reach the 2020 roadmap, as well as the 2030 sustainable development goal (SDG) targets, arguing that these should be set such that programs do not depend disproportionately on foreign aid [6]. In low- and middle-income countries, these targets are less than 0.1% of the domestic health expenditure expected for the period of 2015–2030 [6]. Consequently, economic evaluations are important to help policymakers justify increased domestic health-care funding for LF control.

There have been some estimates of the precontrol health and economic burdens of LF for specific countries/areas [7–9], but no systematic global estimate. This paper aims to estimate the health and economic burdens of LF in GPELF areas prior to MDA programs starting.

## METHODS

A database was created based on data extracted from numerous online sources. This was analyzed and used to calculate the results using Microsoft Excel.

### Epidemiological Model

**Figure 1.**
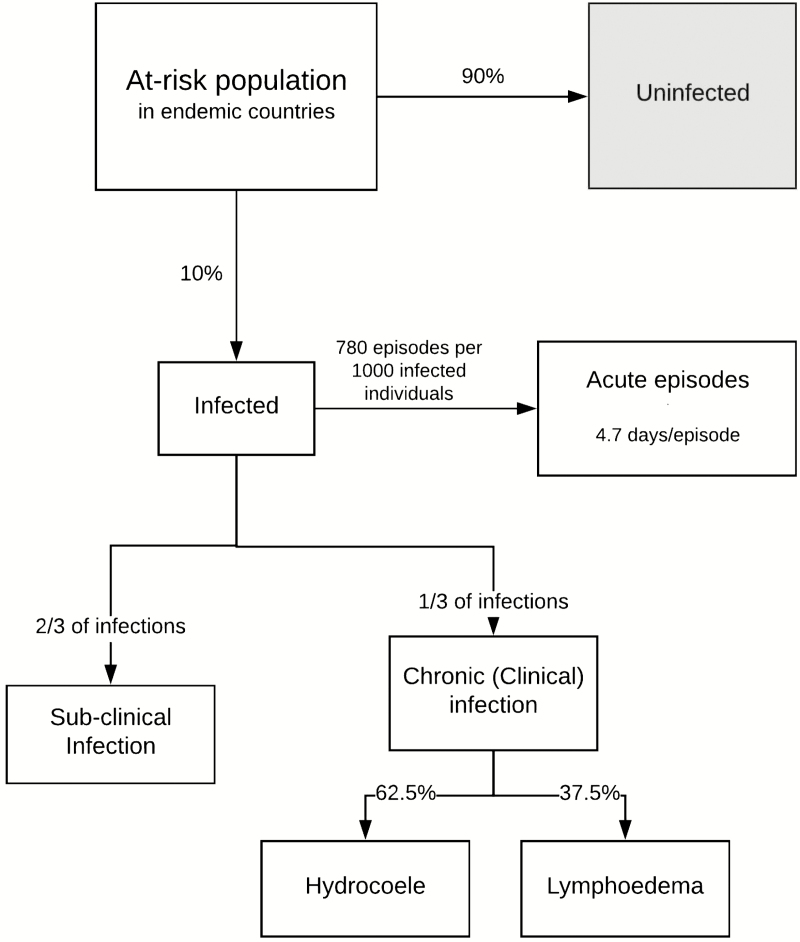
An epidemiological model of lymphatic filariasis. This model was developed based on those of Ottesen et al [10], Chu et al [11], and Turner et al [12].

This research builds on the work of Ottesen et al [10], Chu et al [11], and Turner et al [12]. The epidemiological model (Figure 1) is based on several parameters and assumptions, as described in the Supplementary Information.

Many of these parameters were based on a study by Michael et al [13], which modeled and estimated the global at-risk population of LF to be 1.365 billion across 72 countries and territories (Supplementary Table S1) prior to GPELF, with a total prevalence of 119 million (approximated to 10%), of which 16 million had lymphoedema and 27 million hydrocoele.

### Health Burden

The health burden of LF was summarized in disability-adjusted life years (DALYs): 1 DALY equates to 1 healthy year of life lost. There were 3 different types of clinical morbidity quantified: hydrocoele, lymphoedema, and ADL episodes. The disability weightings were based on those used for LF within the global burden of disease (GBD) 2016 study [14] (Table 1).

**Table 1.** Model Parameters and the Ranges Used Within the Sensitivity Analyses

Parameter	Point Estimate	Min	Max	Sources
**Epidemiological model**				
The proportion of the at-risk population infected	10%	5%	15%	[10, 13, 15]
The proportion of those infected with chronic disease	33.3%	...	...	[10–12]
The proportion of those with chronic disease with hydrocele	62.5%	...	...	[10–12]
The proportion of those with chronic disease with lymphedema	37.5%	*...*	...	[10–12]
Annual incidence of ADL episodes, per 1000 infected individuals	780	*...*	*...*	[16–19]
Average duration of an ADL episode, days	4.7	1	11	[16–19]
**Reductions in productivity**				
During an ADL episode	77.5%	72.5%	84.5%	[20]
With hydrocoele	17.7%	13.8%	20.4%	[20]
With lymphoedema	16.0%	10.0%	21.3%	[20]
**Economic value of a lost productive day**				
GDP per capita of the lowest income quintile	Average: US $2.20^a^	*...*	*...*	[21]
Daily minimum wage (based on USDoS)	Average: US $2.78^a^	*...*	*...*	[22]
Daily minimum wage (based on ILOSTAT)	Average: US$ 2.44^a^	*...*	*...*	[23]
**DALY disability weights**				
ADL disability weight	0.051	0.032	0.074	[14]
Hydrocoele disability weight	0.128	0.086	0.180	[14]
Lymphoedema disability weight	0.109	0.073	0.154	[14]

Parameters relating to treatment seeking are shown in Supplementary Table S3 and Supplementary Figure 1. Costs are in 2016 US$.Abbreviations: ADL, adenolymphangitis; DALY, disability-adjusted life years; GDP, gross domestic product; ILOSTAT, International Labor Organization Statistics; USDoS, United States Department of State.

^a^The economic value of a lost productive day was estimated for each endemic country. The values shown are the global averages (weighted by the countries’ at-risk populations).

### Economic Burden

The economic burden was calculated by quantifying both the direct costs and productivity costs (also known as indirect costs) associated with clinical LF cases. Cost data were adjusted for inflation and standardized to 2016 US$ prices, with the exception of minimum wage data, which was set for a given period.

#### Direct Costs

Direct costs are the costs related to goods, services, and resources consumed to implement and access health care. In this study, direct costs had 2 components: costs incurred by the health-care system in caring for patients, and out-of-pocket costs borne by patients in managing their illnesses. The calculations of these were based on the approach taken by Chu et al [11] and Turner et al [12] (see Supplementary Information).

#### Productivity Costs

Symptomatic LF is debilitating, significantly lowering productivity. Productivity costs represent the value of this loss, and are a product of the reduction in patients’ productivity due to clinical morbidity, based on the value of their time. The productivity losses for LF were quantified using the human capital approach [24], which takes the patient’s perspective for valuing lost productivity and, therefore, counts all the work they missed as a productivity loss.

Our estimates of the reduction in productivity for the various disease states of LF were based on the reported values within a systematic review by Lenk et al [20], of which we took an average (Table 1). When quantifying the number of days with reduced productivity, it was assumed that symptomatic LF cases would have been potentially economically active for 300 days per year, 8 hours a day. Our approach did not differentiate between paid or unpaid lost work (such as household chores or subsistence farming).

Approximating the income/value of time data for individuals with LF is difficult. For example, many of those infected are subsistence farmers, who do not participate in the formal labor market. We therefore estimated the productivity costs using 2 different approaches. For the baseline results, the productivity losses were valued based on the GDP per capita for the lowest income quintile of the population for each country, to adjust for wealth distribution [21] (see Supplementary Information). This approach has been previously used by Redekop et al [25]. Alternatively, we estimated the productivity losses using country-specific daily minimum wages (see Supplementary Information).

Univariate sensitivity analyses were performed on the health and economic burdens and are described in the Supplementary Information.

## RESULTS

### Health Burden

**Figure 2.**
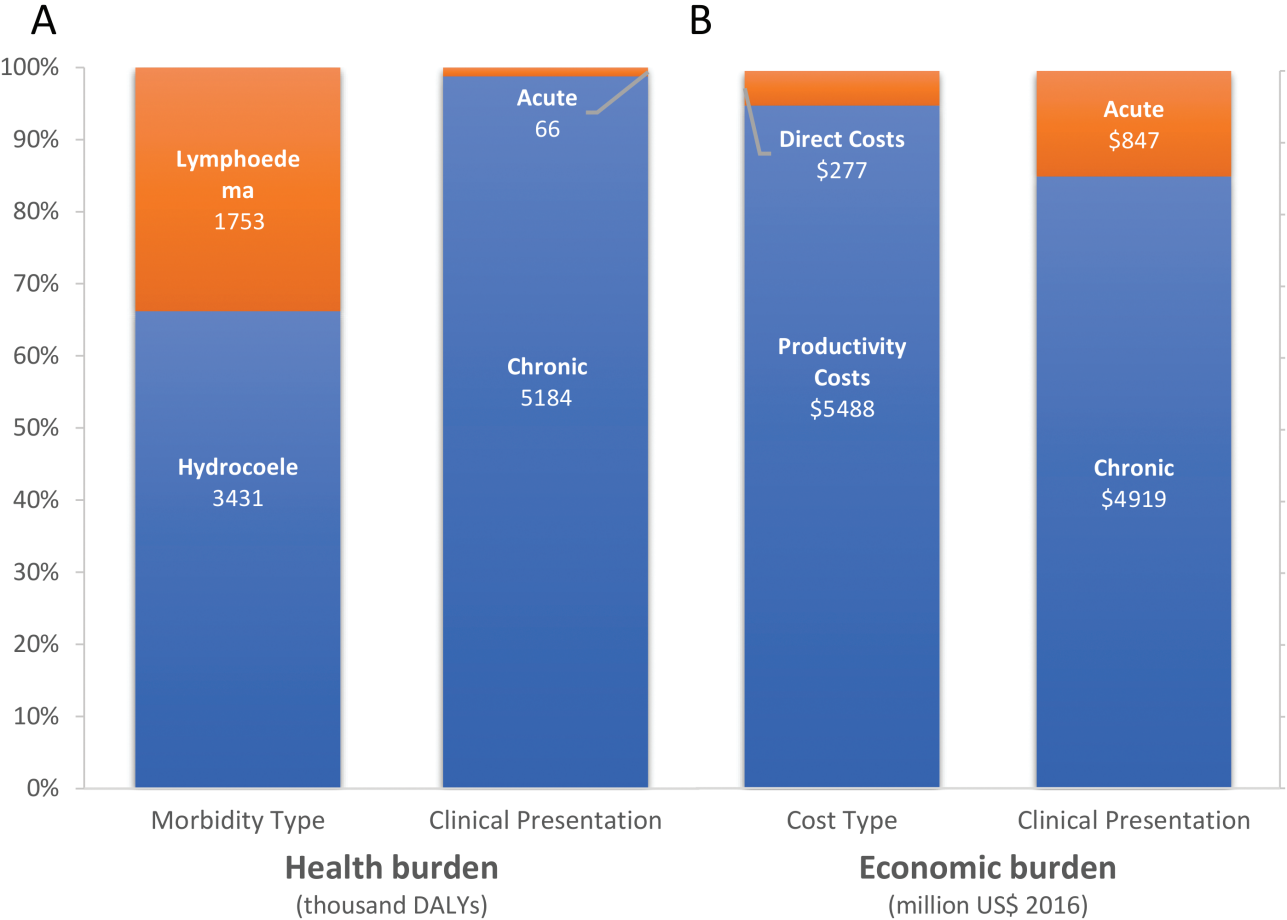
The (*A*) health and (*B*) economic burdens, stratified by morbidity/cost type and clinical presentation. A breakdown of the DALY and economic burden estimates are shown in Supplementary Tables S2, S5, and S7. Results are in 2016 US$. Abbreviation: DALY, disability-adjusted life years.

We estimated that, prior to MDA programs starting, 1.29 billion people were at risk of LF infection in GPELF areas, of which approximately 129 million were infected. Of this population, approximately 86 million people had a subclinical infection and 43 million had a chronic disease (27 million with hydrocoele and 16 million with lymphoedema, with an annual incidence of 100 million ADL episodes amongst all infected; Table 2). This corresponded to a DALY burden of 5.25 million, 99% of which was due to chronic disease, with only 1% from acute ADL episodes (Figure 2; Supplementary Table S2). The WHO South-East Asian and African regions accounted for the great majority of this burden (61% and 32%, respectively), with India, Indonesia, and Nigeria being the most heavily burdened countries.

**Table 2.** The Estimated Health and Economic Burdens, Stratified by Region

Region	Number at Risk of Infection, Millions	Number Infected, Millions	Number With Hydrocoele, Millions	Number With Lymphoedema, Millions	Annual Incidence of ADL Episode, Millions	DALY Burden, Millions	Average Economic Burden Per Chronic Case	Total Economic Burden, Millions
AFRO	416.90	41.69	8.69	5.21	32.52	1.70	$67.40	$1148
EMRO	23.83	2.38	0.50	0.30	1.86	0.10	$145.87	$134
PAHO	8.87	0.89	0.18	0.11	0.69	0.04	$111.85	$40
SEARO	789.98	79.00	16.46	9.87	61.62	3.22	$135.47	$4137
WPRO	47.04	4.70	0.98	0.59	3.67	0.19	$169.53	$307
Total	1286.62	128.66	26.80	16.08	100.36	5.25	$114.69	$5765

Costs are in 2016 US$.Abbreviations: ADL, adenolymphangitis; AFRO, WHO African region; DALY, disability-adjusted life years; EMRO, WHO Eastern Mediterranean region; PAHO, WHO pan-American region; SEARO, WHO South-East Asian region; WHO, World Health Organization; WPRO, WHO Western Pacific region.

### Economic Burden

Prior to MDA programs starting, the total economic burden of LF was estimated to be US $5.765 billion annually (when using the GDP per capita of the lowest income quintile of the population to value productivity losses; Table 2; Supplementary Table S4). The direct costs to the health-care system contributed US $0.155 billion (2.7%), patient medical expenses contributed US $0.122 billion (2.1%), and productivity losses contributed US $5.488 billion (95.2%; Supplementary Tables S5 and S7). We estimated that 2.5 billion productive days would be lost per year due to LF (Supplementary Table S6).

This estimate varies depending on how the productivity losses were valued. For example, when minimum wage data was used, then the burden ranged from US $6.372 billion (International Labor Organization Statistics data) to US $7.210 billion (United States Department of State data; Supplementary Table S8). The annual direct costs of LF, which consists of health system costs and out-of-pocket patient expenses, totalled US $0.277 billion. Of these costs, 68% were due to ADL episodes (Supplementary Table S7), while ADL episodes only accounted for 12% of productivity costs.

The average annual economic burden per chronic LF case was estimated to be US $114.69 (Supplementary Table S9), the majority of which resulted from lost productivity costs (US $112.62). US $0.87 per case annually can be attributed to out-of-pocket costs, and US $1.20 to the cost to the health-care system. On average, chronic LF cases lost 51 productive days per year.

As with the health burden, the WHO South-East Asian (73%) and African (19%) regions contained the majority of the economic burden.

### Sensitivity Analyses

As expected, the health and economic burdens were most sensitive to the proportion of the at-risk population infected (*Supplementary Figure S2A*and*B*). The variation in the disability weightings of hydrocoele, lymphoedema, and ADLs also had a direct effect on the number of DALYs estimated, mirroring the proportion of DALYs attributable to each of the sequelae. The effect of varying the duration of ADL episodes was minimal (Supplementary Figure S2*A*).

Apart from infected population, the economic burden is most sensitive to the productivity reductions due to ADL episodes, lymphoedema, and hydrocoele (Supplementary Figure S2*B*). The parameters related to treatment-seeking behavior (Supplementary Figure S1) and the ADL episode incidence had no notable impacts. The total economic burden ranged from US $1.499–13.236 billion, with the parameters minimized and maximized, respectively.

## Discussion

Prior to MDA programs starting, our model indicated that LF was responsible for 5.25 million DALYs and 2.496 billion lost productive days annually. The estimated economic burden varied depending on the calculation method (Table 2). When using GDP per capita of the lowest income quintile of the population, the burden was US $5.765 billion, but this increased to US $7.210 billion when using minimum wage data from the United States Department of State. The productivity costs accounted for the majority of the economic burden (95.2%). These results clearly demonstrate the magnitudes of both the health and economic burdens of LF. Using a similar framework, Turner et al [12] projected that, due to the first 15 years of the GPELF, potentially 175 million DALYs have been averted and US $100.5 billion saved over the lifetimes of those who received treatment. Again, the majority of this economic benefit was from prevented productivity costs [12].

The third WHO NTD report estimated that at least US $154 million is needed per year from 2015–2020 for MDA for LF alone, which excludes the additional investments needed for surveillance and morbidity management after transmission is eliminated [6]. Our findings support the justification for continued investment for LF control, as well as governments increasing their domestic spending on control and morbidity management programs.

While achieving elimination of LF transmission is critical to achieving SDG 3 (“ensure healthy lives and promote wellbeing for all at all ages” [26]), scaling up morbidity management programs is also vital when considering the burden of the remaining chronic clinical disease of LF. Elimination and morbidity management are also significant in several other SDGs, such as SDGs 1 and 2, given the economic burden associated with clinical LF [26].

Other studies have also found that LF is associated with high productivity costs [7]. For example, it has been estimated that, in India, between 3.8–8% of the potential male labor input was lost due to chronic LF morbidity [7], for an estimated productivity cost of US $704 million per year (in 1995 prices) [8]. A similar value has been reported for Ghana, where over 7% of potential male labor was estimated to be lost due to chronic LF [27].

Understanding this precontrol disease burden is vital as, even if MDA eliminates LF transmission, many with chronic morbidity will remain [15]. These individuals will continue to experience notable health and economic burdens due to LF (on average US $114.62 per year) further highlighting the need for continued morbidity management programs, which are currently only established in 53% of endemic countries [4]. Studies have found that morbidity management can be cost effective and generate notable economic benefits [7, 28, 29].

### Uncertainty Surrounding the Economic Burden of Lymphatic Filariasis and Productivity Costs

The baseline estimate of the economic burden of LF was US $5.765 billion and ranged from US $1.499–13.236 billion, when all parameters were minimized and maximized, respectively (Table 1; Supplementary Table S3; Supplementary Figure S2). The productivity costs accounted for the clear majority of this estimated burden, and it is therefore important to carefully evaluate how these were calculated.

#### Quantifying Productivity Losses

The assumed productivity losses associated with clinical LF were based on studies identified by a systematic literature review [20]. The majority of the studies focused on quantifying the proportion of work missed by LF patients (known as absenteeism [24]). However, in addition to working fewer hours/days, LF patients may also be less productive while at work due to their illness (known as presenteeism [24]). Not accounting for this can underestimate the estimated productivity losses due to clinical LF [11]: for example, Ramu et al [30] found that though the reported time difference (hours worked) between LF infected and uninfected weavers was 15–20%, the actual productivity gap was higher, at 27%. The studies also have focused on the productivity losses of the LF patients; however, their informal caregivers could also have experienced productivity losses, which were not accounted for in this study.

We used the human capital approach to estimate the productivity losses. This takes the patient’s perspective for valuing lost productivity and counts all missed work as a productivity loss. However, this has been criticized for overestimating productivity costs, as it typically assumes that time lost by an individual in the labor market is not compensated for by an otherwise unproductive person: that is, it does not account for the fact that an ill employee will eventually be replaced [24, 31]. An alternative method, known as the friction cost approach, takes the employer’s perspective for valuing lost productivity, and only counts the hours not worked by a sick employee before another employee takes over the work [31]. This can result in much lower estimates of productivity losses/costs, and there is continued debate within the field regarding which approach is most appropriate [24]. In the context of studies for NTDs, the friction cost approach is difficult to apply, as the majority of those infected are not in formal employment.

#### Valuing Productivity Losses

Accurately valuing productivity losses is never simple, particularly for NTDs, where the majority of those infected do not participate in the formal labor market. A variety of methods have been used in economic analyses of similar tropical diseases (such as minimum wages, the average value added per agricultural worker, and proxies from prior studies in similar settings) [7, 32].

For our baseline results, we used the GDP per capita of the lowest income quintile of the population. A limitation of this is that in some settings (such as urban areas), this could underestimate LF-related productivity costs. Using the minimum wage to value productivity losses was an alternative approach we considered. However, several countries do not have a prescribed minimum wage, and in many that do, it is not always actively enforced (particularly in rural areas), nor applicable in the informal labor market. Additionally, the values are often poorly publicized, with sources providing notably different values for the same country. More socioeconomic research is necessary to yield greater accuracy regarding the economic burden of LF (and other NTDs).

We assumed that clinical LF cases would have been potentially economically active for 300 days per year, 8 hours a day (accounting for both paid and unpaid work). We did not specifically value lost leisure time, due to the challenges in distinguishing between unpaid labor and leisure time [24].

### Limitations

The estimates of health and economic burdens of diseases can vary considerably, due to differences in research data, methodology, and context. An example is the variation in baseline assumptions between research, which, while similar, were still notably different, and thus could have contributed to differences in results. Beyond this, among the largest limitations of this study is the uncertainty surrounding the precontrol prevalence of LF. The estimates we used were based on the number at risk of infection, as recorded by the Preventive Chemotherapy and Transmission Control (PCT) databank, and the assumption that, on average, 10% of those at risk will be infected (resulting in an estimated precontrol burden of 129 million people infected and 5.25 million DALYs). However, current GBD study estimates of the prevalence of LF and its corresponding health burden in 1999, prior to the launch of GPELF, were significantly lower than this (52 million infected and 1.9 million DALYs) [33]. Within the GBD study, LF infection prevalence estimates were modeled using DisMod-MR 2.1, informed by data in the PCT Databank. The lymphoedema and hydrocoele prevalence estimates were also modeled using DisMod-MR 2.1, based on data from the Global Atlas of Helminth Infections. Part of the difference may be due to how coinfections are accounted for within the GBD estimates; however, without additional information it is difficult to examine this variation further. A lower average prevalence, in line with the GBD estimates, was considered in our sensitivity analysis.

The assumptions that, on average, 10% of the at-risk population are infected, of which one-third bear chronic infections, were based on a review by Michael et al [13]. Given that this review was conducted over 20 years ago, it is justifiable to assume these proportions would be applicable when considering the at-risk populations in GPELF countries prior to MDA programs. However, our model assumed that the above proportions could be applied uniformly to the at-risk populations, which ignores the varying distribution of disease. In reality, LF demonstrates significant heterogeneity in its transmission dynamics [34].

It should be noted that, although the economic evaluations of the GPELF are often based on very similar assumptions, there are at times differences in the assumed number at risk and the number of countries covered by the GPELF.

Due to a lack of regional/country-specific data, many of the parameters (eg, ADL frequency and duration, work hours lost) were attributed to a global standardized estimate. Although much of the literature originated from India and sub-Saharan African countries (where the majority of the at-risk population resides), the sensitivity analysis included a range of different values and the overall results appear robust.

Our analysis focused on GPELF areas. However, LF was also previously endemic in some non-GPELF countries (such as China), and the burden in these areas was not captured within our analysis.

Due to the absence of data, no excess mortality of clinical patients was assumed. However, this could be underestimating the DALY burden of LF.

## CONCLUSION

Prior to MDA programs starting, we estimate that LF contributed to 5.25 million DALYs, amounting to an economic burden of US $5.765 billion annually. These results clearly demonstrate the magnitudes of both the health and economic burdens of LF in the absence of control, and highlight the continued need to support the GPELF. We found that chronic patients themselves bore the majority of the economic burden (US $114.69 per chronic case), despite being the least able to afford these losses. This further highlights the need to scale up LF morbidity management programs to prevent individuals with clinical disease from being left behind.

## Supplementary Data

Supplementary materials are available at *Clinical Infectious Diseases* online. Consisting of data provided by the authors to benefit the reader, the posted materials are not copyedited and are the sole responsibility of the authors, so questions or comments should be addressed to the corresponding author.

ciz671_suppl_Supplementary_Material

## Notes

***Author contributions***. C. G. M. and H. C. T. conceived the study and wrote the first draft. C. G. M. coded the model and analyzed the data. B. K. C., E. A. O., and M. H. B. designed the original modeling framework. All authors contributed to the writing. All authors read and approved the final version of the manuscript.

***Acknowledgments.*** The authors thank Guy E. Thwaites for valuable comments on the manuscript.

***Potential conflicts of interest*.** H. C. T. is supported by the Wellcome Trust (grant number 089276/B/09/7). C. G. M. was financially supported by the ExxonMobil Global Health Scholarship. M. H. B. is employed by GlaxoSmithKline, which donates albendazole to the World Health Organization for use in the global lymphatic filariasis elimination initiative. All other authors report no potential conflicts. All authors have submitted the ICMJE Form for Disclosure of Potential Conflicts of Interest. Conflicts that the editors consider relevant to the content of the manuscript have been disclosed.
