# Supplementary material for: The Health and Economic Burdens of Lymphatic Filariasis Prior to Mass Drug Administration Programs
Source: Clin Infect Dis. 2019 Jul 25;70(12):2561–7. doi: 10.1093/cid/ciz671 (PMC7286370; doi:10.1093/cid/ciz671)
Supplement: ciz671_suppl_Supplementary_Material [file ciz671_suppl_supplementary_material.docx]

**Supporting Information**

**At-risk population**

The at-risk populations were primarily based on data from the WHO’s PCT Databank [13]. The population at-risk would be those living in regions endemic to LF within each country, which is not necessarily equivalent to the country’s entire population. Estimates were either chosen based on the at-risk population in the year prior to the start of MDA for each country, or the first available estimate following completion of a national LF mapping exercise. If mapping in the country was incomplete at this point, then the estimates before and after completion of mapping were compared and the higher of the two values was used. Given this, the reference year for each country’s at-risk population will differ depending on the varying starts of their respective MDA programmes.

| Supporting Table S1. At-Risk Population in Countries Endemic for LF Prior to MDA Under GPELF | | | | | |
| --- | --- | --- | --- | --- | --- |
| Countries/territories | **Country code** | **WHO region** | **World Bank classification** | **At-risk population** | Year of estimate |
| Angola | *AGO* | *AFRO* | *LMIC* | 12,090,000 | 2015 |
| American Samoa | *ASM* | *WPRO* | *UMIC* | 63,000 | 2000 |
| Burundi | *BDI* | *AFRO* | *LIC* | - | - |
| Benin | *BEN* | *AFRO* | *LIC* | 3,430,000 | 2002 |
| Burkina Faso | *BFA* | *AFRO* | *LIC* | 12,000,000 | 2001 |
| Bangladesh | *BGD* | *SEARO* | *LMIC* | 34,000,000 | 2001 |
| Brazil | *BRA* | *PAHO* | *UMIC* | 1,500,000 | 2004 |
| Brunei Darussalam | *BRN* | *WPRO* | *HIC* | 15,000 | 2013 |
| Central African Republic | *CAF* | *AFRO* | *LIC* | 3,300,000 | 2009 |
| Côte d'Ivoire | *CIV* | *AFRO* | *LMIC* | 14,000,000 | 2009 |
| Cameroon | *CMR* | *AFRO* | *LMIC* | 14,305,000 | 2008 |
| Democratic Republic of the Congo | *COD* | *AFRO* | *LIC* | 49,140,000 | 2013 |
| Congo | *COG* | *AFRO* | *LMIC* | 2,600,000 | 2009 |
| Cook Islands | *COK* | *WPRO* | *#N/A* | 20,000 | 2000 |
| Comoros | *COM* | *AFRO* | *LIC* | 456,300 | 2001 |
| Cape Verde | *CPV* | *AFRO* | *LMIC* | - | - |
| Costa Rica | *CRI* | *PAHO* | *UMIC* | - | - |
| Dominican Republic | *DOM* | *PAHO* | *UMIC* | 740,000 | 2002 |
| Egypt | *EGY* | *EMRO* | *LMIC* | 2,100,000 | 2000 |
| Eritrea | *ERI* | *AFRO* | *LIC* | 69,600 | 2015 |
| Ethiopia | *ETH* | *AFRO* | *LIC* | 30,000,000 | 2009 |
| Fiji | *FJI* | *WPRO* | *UMIC* | 841,500 | 2002 |
| Micronesia, Federated States of | *FSM* | *WPRO* | *LMIC* | 114,100 | 2003 |
| Gabon | *GAB* | *AFRO* | *UMIC* | 1,290,600 | 2009 |
| Ghana | *GHA* | *AFRO* | *LMIC* | 4,000,000 | 2000 |
| Guinea | *GIN* | *AFRO* | *LIC* | 6,067,100 | 2013 |
| Gambia | *GMB* | *AFRO* | *LIC* | 1,200,000 | 2014 |
| Guinea-Bissau | *GNB* | *AFRO* | *LIC* | 1,311,700 | 2010 |
| Equatorial Guinea | *GNQ* | *AFRO* | *UMIC* | 420,000 | 2016 |
| Guyana | *GUY* | *PAHO* | *UMIC* | 630,000 | 2003 |
| Haiti | *HTI* | *PAHO* | *LIC* | 6,000,000 | 2001 |
| Indonesia | *IDN* | *SEARO* | *LMIC* | 124,574,000 | 2009 |
| India | *IND* | *SEARO* | *LMIC* | 553,680,000 | 2005 |
| Kenya | *KEN* | *AFRO* | *LMIC* | 2,987,300 | 2002 |
| Cambodia | *KHM* | *WPRO* | *LMIC* | 435,000 | 2005 |
| Kiribati | *KIR* | *WPRO* | *LMIC* | 95,200 | 2001 |
| Lao People's Democratic Republic | *LAO* | *WPRO* | *LMIC* | 117,600 | 2009 |
| Liberia | *LBR* | *AFRO* | *LIC* | 3,600,000 | 2011 |
| Sri Lanka | *LKA* | *SEARO* | *LMIC* | 9,000,000 | 2001 |
| Madagascar | *MDG* | *AFRO* | *LIC* | 15,821,700 | 2005 |
| Maldives | *MDV* | *SEARO* | *UMIC* | 1,900 | 2004 |
| Marshall Islands | *MHL* | *WPRO* | *UMIC* | 900 | 2003 |
| Mali | *MLI* | *AFRO* | *LIC* | 11,408,300 | 2005 |
| Myanmar | *MMR* | *SEARO* | *LMIC* | 45,675,700 | 2006 |
| Mozambique | *MOZ* | *AFRO* | *LIC* | 15,538,600 | 2009 |
| Mauritius | *MUS* | *AFRO* | *UMIC* | - | 2003 |
| Malawi | *MWI* | *AFRO* | *LIC* | 12,887,200 | 2008 |
| Malaysia | *MYS* | *WPRO* | *UMIC* | 1,169,600 | 2003 |
| New Caledonia | *NCL* | *WPRO* | *HIC* | 12,400 | 2009 |
| Niger | *NER* | *AFRO* | *LIC* | 10,555,300 | 2007 |
| Nigeria | *NGA* | *AFRO* | *LMIC* | 106,124,900 | 2011 |
| Niue | *NIU* | *WPRO* | *#N/A* | 2,000 | 2000 |
| Nepal | *NPL* | *SEARO* | *LIC* | 22,000,000 | 2003 |
| Philippines | *PHL* | *WPRO* | *LMIC* | 36,000,000 | 2000 |
| Palau | *PLW* | *WPRO* | *HIC* | 20,000 | 2009 |
| Papua New Guinea | *PNG* | *WPRO* | *LMIC* | 5,820,000 | 2005 |
| French Polynesia | *PYF* | *WPRO* | *HIC* | 226,000 | 2000 |
| Rwanda | *RWA* | *AFRO* | *LIC* | - | - |
| Sudan | *SDN* | *EMRO* | *LMIC* | 19,893,800 | 2013 |
| Senegal | *SEN* | *AFRO* | *LIC* | 4,248,600 | 2007 |
| Solomon Islands | *SLB* | *WPRO* | *LMIC* | - | - |
| Sierra Leone | *SLE* | *AFRO* | *LIC* | 4,967,800 | 2007 |
| South Sudan | *SSD* | *EMRO* | *LIC* | 1,659,600 | 2016 |
| São Tomé and Príncipe | *STP* | *AFRO* | *LMIC* | 188,100 | 2016 |
| Suriname | *SUR* | *PAHO* | *UMIC* | - | - |
| Seychelles | *SYC* | *AFRO* | *HIC* | - | - |
| Chad | *TCD* | *AFRO* | *LIC* | 7,270,000 | 2009 |
| Togo | *TGO* | *AFRO* | *LIC* | 1,600,000 | 2000 |
| Thailand | *THA* | *SEARO* | *UMIC* | 125,700 | 2002 |
| Timor-Leste | *TLS* | *SEARO* | *LMIC* | 924,600 | 2005 |
| Tonga | *TON* | *WPRO* | *UMIC* | 98,000 | 2001 |
| Trinidad and Tobago | *TTO* | *PAHO* | *HIC* | - | - |
| Tuvalu | *TUV* | *WPRO* | *UMIC* | 9,600 | 2001 |
| Tanzania (incl. Zanzibar) | *TZA* | *AFRO* | *LIC* | 35,348,700 | 2000 |
| Uganda | *UGA* | *AFRO* | *LIC* | 12,429,400 | 2002 |
| Viet Nam | *VNM* | *WPRO* | *LMIC* | 1,480,000 | 2003 |
| Vanuatu | *VUT* | *WPRO* | *LMIC* | 196,200 | 2000 |
| Wallis and Futuna | *WLF* | *WPRO* | *#N/A* | 15,000 | 2002 |
| Samoa | *WSM* | *WPRO* | *UMIC* | 286,300 | 2006 |
| Yemen | *YEM* | *EMRO* | *LMIC* | 173,300 | 2002 |
| Zambia | *ZMB* | *AFRO* | *LMIC* | 8,780,000 | 2012 |
| Zimbabwe | *ZWE* | *AFRO* | *LIC* | 7,466,600 | 2015 |
| *AFRO: WHO African Region, EMRO: WHO Eastern Mediterranean Region, PAHO: WHO Pan-American Region, SEARO: WHO South-East Asian Region, WPRO: WHO Western Pacific Region, LIC: Low Income Country, LMIC: Lower Middle-Income Country, UMIC: Upper-Middle Income Country, HIC: High Income Country.* | | | | | |

**Incidence of ADL episodes**

Within Chu et al. [[1](#_ENREF_1)] and Turner et al. [[2](#_ENREF_2)] it was assumed that a negligible proportion of the subclinical population experienced ADLs, while 70% of hydrocoele and 95% of lymphoedema patients experienced an average of two and four ADL episodes annually respectively, lasting four days each. However, based on [[3-6](#_ENREF_3)] this assumption was updated and we assumed an average incidence of ADLs of 780 episodes per 1,000 infected individuals, with an average duration of 4.7 days per an episode.

**Parameters and Assumptions of the Epidemiological Model**

The epidemiological model (Figure 1) is based on the following parameters and assumptions:

- 72 countries and territories are covered by the GPELF (Supporting Table S1) [[12](#_ENREF_12)]*.*
- On average 10% of the at-risk population will develop infection [[9](#_ENREF_9), [13](#_ENREF_13), [14](#_ENREF_14)].
- Two-thirds of those infected will develop subclinical infection and one-third symptomatic, chronic clinical infection [[9](#_ENREF_9)].
- 62.5% of symptomatic infections develop hydrocoele and 37.5% lymphedema [[9](#_ENREF_9)].
- Those with infection may experience ADL episodes, with an average incidence of 780 episodes per 1,000 infected individuals per year (see supporting information).

**The Value and Limitations of Disability-Adjusted Life Years**

We estimated that pre-control LF accounted for 5.25 million DALYs (ranging between 1.393 – 11.368 million based on the 95% CI for the disability weights (Table 1)) annually. Although this is notable, it is important to consider that DALYs make significant implicit assumptions and have limitations.

The universal DALY disability weights do not account for how the local context may influence the burden of a disease, and in particular, the fact that the burden may be worse for those who are living in poverty [[26](#_ENREF_26)]. DALYs also have shortcomings regarding accounting for the psycho-social impact of illness on the individual and their quality of life. Ton et al. [[27](#_ENREF_27)], highlighted that the DALY burden attributable to LF could increase significantly if depression experienced by LF patients is also quantified. In addition, DALYs primarily consider health burden in terms of what is directly experienced by the person bearing the illness and fail to account for the burden experienced by their caregivers [[27](#_ENREF_27)].

While DALYs may have many shortcomings, they still provide an objective and standardised method for quantifying disease burden and remain the gold standard measure. Interestingly, the DALY weights poorly relate to the estimated productivity losses (Table 1).

While a proportion of the patients experiencing ADL episodes would concurrently also have lymphoedema or hydrocele, the effect of co-morbid disease on the DALY estimate was assumed to be negligible. Due to the absence of data, no excess mortality of clinical patients was assumed. Based on the methodology employed since the GBD 2010 study, we did not apply a discount rate or age weighting to the DALY estimates.

| Supporting Table S2. Disability adjusted life years attributable to sequelae of LF prior to GPELF | | | | |
| --- | --- | --- | --- | --- |
| **Region** | **Hydrocoele (thousands)** | **Lymphoedema (thousands)** | **ADL episodes (thousands)** | **Total**  **(thousands)** |
| **AFRO** | 1,111.74 | 568.03 | 21.29 | 1,701.06 |
|  |  |  |  | *32.4%* |
| **EMRO** | 63.54 | 32.46 | 1.22 | 97.22 |
|  |  |  |  | *1.9%* |
| **PAHO** | 23.65 | 12.09 | 0.45 | 36.19 |
|  |  |  |  | *0.7%* |
| **SEARO** | 2,106.62 | 1,076.35 | 40.34 | 3,223.31 |
|  |  |  |  | *61.4%* |
| **WPRO** | 125.43 | 64.09 | 2.40 | 191.92 |
|  |  |  |  | *3.7%* |
| **Total** | **3,430.98** | **1,753.02** | **65.69** | **5,249.70** |
|  | *65.4%* | *33.4%* | *1.3%* |  |
| *AFRO: WHO African Region, EMRO: WHO Eastern Mediterranean Region, PAHO: WHO Pan-American Region, SEARO: WHO South-East Asian Region, WPRO: WHO Western Pacific Region, ADL: Acute Adenolymphangitis.* | | | | |

### **Direct Costs**

The direct costs within this this study involve two components: the costs faced by the healthcare system in treating symptomatic patients, and costs borne by patients in managing their illness (see [[1](#_ENREF_1), [2](#_ENREF_2)]).

Healthcare system costs

The direct costs incurred by the healthcare system costs are a result of patients interacting with the healthcare system and seeking medical care for their illness. We have assumed this to be equivalent to the cost of an outpatient visit to a rural public health facility (more conservative than an urban facility), as reported in the WHO-CHOICE database with estimates on the unit costs for service delivery [[7](#_ENREF_7)]. In its most recent version (2011), this database provides unit costs for most countries for 2008. The overall cost is thus a product of the number of symptomatic infections seeking care in a public facility and the cost per an outpatient visit to a rural facility (Supporting Figures S1). Due to the absence of data regarding the cost and actual number of hydrocele surgeries performed in endemic countries before the GEPLF, it was not possible to incorporate their costs into the analysis.

Patient’s medical expenses

When considering the patient’s medical expenses, the treatment patients make use of can be split into two groups: those that do not get treated, and those that do. Of those that do, there are three types of costs to evaluate – the costs of receiving treatment in a public facility, treatment in a private facility, or self-treatment. The costs of utilising a facility consists of the cost of medication, the doctor’s fee and other costs such as travel. Treatment with medication includes analgesia (ibuprofen), antipyretics (paracetamol) and in the case of ADLs, antibiotics (penicillin e.g. amoxicillin) [[8](#_ENREF_8), [9](#_ENREF_9)]. Those that self-treat face lower costs as they do not receive antibiotics nor do they visit a facility. These costs would also differ slightly between patients suffering from acute episodes and those only with chronic symptoms (Supporting Figure S1), of which we assumed a larger proportion will self-treat. Those that self-treat would not interact with the healthcare system and thus no healthcare system costs would be attributed to these treatments. Due to the availability of data regarding health-seeking behaviour in LF patients in India, we were able to use custom parameters for India.

The cost of medication is assumed to be equivalent to treatment for a week (seven days) with conservative use of three medications: amoxicillin (500mg three times a day), paracetamol (500mg three times a day) and ibuprofen (400mg twice a day) [[10](#_ENREF_10)]. This improves on the calculations of Chu *et al.* [[1](#_ENREF_1)] and Turner *et al.* [[2](#_ENREF_2)], who assumed a single daily dose of each of the medications. Their costs were calculated based on prices from the International Medical Products Price Guide produced by Management Sciences for Health (MSH) [[11](#_ENREF_11)]. The most recent Median Price Ratios for each country were used, with the lowest regional value being used for countries with no data. The cost of doctor’s fees and other costs associated with utilising healthcare are assumed to be equivalent to 60% and 40% of the cost of the medication– as was assumed by previous analyses [[1](#_ENREF_1), [2](#_ENREF_2)]. Supporting Table S3 describes the treatment seeking parameters.

In instances where data for the healthcare system costs or patient’s medical expenses were not available for a country, the lowest value found in that WHO region was used as a proxy. While it can be argued that this would allow for significant underestimation, this was done to ensure the estimates remained conservative.

The relevant cost data were adjusted for inflation and standardised to 2016 prices. This was done by using the relevant countries GDP deflator (i.e. using the local countries inflation rates).

| **Supporting Table S3: Treatment seeking parameters** | | | | |
| --- | --- | --- | --- | --- |
| **Parameter** | **Point estimate** | **Min** | **Max** | **Sources** |
| **Percentage seeking treatment** |  |  |  |  |
| Seeking treatment with hydrocoele | 30% | 20% | 80% | [[5](#_ENREF_5), [12-15](#_ENREF_12)] |
| *India*^1^ | 60% | 41% | 80% |  |
| Seeking treatment with lymphoedema | 35% | 30% | 100% |  |
| *India*^1^ | 65% | 45% | 100% |  |
| **Frequency of seeking treatment** | | | | |
| Frequency for ADL (per year per infected) | 0.43 | 0.39 | 1.56 | [[5](#_ENREF_5), [12-15](#_ENREF_12)] |
| *India*^1^ | 0.59 | 0.21 | 1.56 |  |
| Frequency for hydrocoele (per year) | 2 | 1 | 4 |  |
| Frequency for lymphoedema (per year) | 3 | 1 | 6 |  |
| **Treatment Preferences** | **Public** | **Private** | **Self-Treat** |  |
| For patients experiencing ADL Episodes | 25% | 5% | 70% | [[5](#_ENREF_5), [12-15](#_ENREF_12)] |
| *India*^1^ | 55% | 35% | 10% |  |
| For hydrocoele/lymphoedema patients | 15% | 5% | 80% |  |
| *India*^1^ | 55% | 35% | 10% |  |
| ^1^ *Specific parameters for India are listed as data is available at a regional level for the Indian context.* | | | | |

**Supporting Figure S1. Treatment seeking behaviour of LF patients*.*** *The sources of the parameters are outlined in Supporting Table S3.* ^1^*Specific parameters for India are listed as data is available at a regional level for the Indian context.*

75%^1^

60%^1^

65%^1^

Treatment Seeking in India

Direct Patient Medical Expenses

Direct Health System Costs

ADL Patients

Public Facility

Private Facility

Self-Treatment

No Treatment

1. Medication:
   - Paracetamol
   - Ibuprofen
2. Medication:
   - Amoxicillin
   - Paracetamol
   - Ibuprofen
3. Doctor’s fees (for private)
4. Other e.g. transport

55%^1^

10%^1^

35%^1^

25%

25%

70%

5%

55% Seek Treatment

Cost of Outpatient Visit

Hydrocoele Patients

Public Facility

Private Facility

Self-Treatment

No Treatment

1. Medication:
   - Paracetamol
   - Ibuprofen
2. Medication:
   - Amoxicillin
   - Paracetamol
   - Ibuprofen
3. Doctor’s fees (for private)
4. Other e.g. transport

55%^1^

10%^1^

35%^1^

70%

15%

80%

5%

30% Seek Treatment

Cost of Outpatient Visit

Lymphoedema Patients

Public Facility

Private Facility

Self-Treatment

No Treatment

1. Medication:
   - Paracetamol
   - Ibuprofen
2. Medication:
   - Amoxicillin
   - Paracetamol
   - Ibuprofen
3. Doctor’s fees (for private)
4. Other e.g. transport

55%^1^

10%^1^

35%^1^

65%

15%

80%

5%

35% Seek Treatment

Cost of Outpatient Visit

Productivity costs

The productivity of each individual was valued according to their contribution to the GDP. Given the disproportionate effect of LF on impoverished populations, and for conservative purposes, this was assumed to be the GDP generated by the lowest quintile of the population. Using the income distribution for each country, and the proportion attributed to the lowest quintile, the GDP generated by that segment of the population was used to calculate a daily value of productivity as per the equation below, with an assumed 300 productive days per a year.

$$Daily Value of Productivity=\frac{Annual Gross Domestic Product per Capita}{Productive Days in a Year}\times\frac{Income Distribution for Lowest Quintile}{20\%}$$

The minimum wage rates were standardised to daily rates by assuming eight-hour working days, five work days a week, for 52 weeks, amounting to 260 working days in a year. This daily rate was then applied to a total of 300 productive days per year to partly account for unpaid work that is not covered in the 260 days. Similarly, the GDP was assumed to be generated over 300 productive days in a year.

The values are summarised in Supporting Table S4.

In instances where GDP or minimum wage data was not available for a country, the weighted (according to at-risk population) average for that WHO region was used as a proxy.

We considered two different sources for the minimum wage data: the United States Department of State and the International Labour Organisation (Supporting Table S4) [[20](#_ENREF_20), [21](#_ENREF_21)]. For countries where data for these wage sources were not available, the weighted (according to at-risk population) average for the GPELF countries in that WHO region was used.

| Supporting Table S4: Estimated economic value of a productive day | | | |  |
| --- | --- | --- | --- | --- |
| **Region** | **Based on the minimum wage** | | **Based on GDP per capita of the lowest income quintile [**[**16**](#_ENREF_16)**]** | |
|  | **USDoS^1^ [**[**17**](#_ENREF_17)**]** | **ILOSTAT [**[**18**](#_ENREF_18)**]** |  |  |
| **AFRO** | US$2.75 | US$2.73 | US$1.82 | |
| **EMRO** | US$2.77 | US$1.88 | US$2.51 | |
| **PAHO** | US$7.24 | US$7.83 | US$5.86 | |
| **SEARO** | US$4.17 | US$4.43 | US$4.16 | |
| **WPRO** | US$8.97 | US$5.93 | US$3.51 | |
| **Weighted Average** | **US$2.78** | **US$2.44** | **US$2.20** | |
| *AFRO: WHO African Region, EMRO: WHO Eastern Mediterranean Region, PAHO: WHO Pan-American Region, SEARO: WHO South-East Asian Region, WPRO: WHO Western Pacific Region, USDoS: United States Department of State, ILOSTAT: International Labour Organization Statistics.*  *The values are weighted by the at-risk population of the endemic countries.*  *^1^In some cases where data was incomplete, it was necessary to combine this with other online databases and sources.* | | | | |

| Supporting Table S5: Productivity costs by WHO region | | | | |
| --- | --- | --- | --- | --- |
| **Region** | **Hydrocoele  (US$ thousands)** | **Lymphoedema  (US$ thousands)** | **ADL episodes  (US$ thousands)** | **Total  (US$ thousands)** |
| **AFRO** | 583,343 | 317,359 | 122,735 | 1,023,437 |
|  |  |  |  | *18.6%* |
| **EMRO** | 74,141 | 40,336 | 15,599 | 130,076 |
|  |  |  |  | *2.4%* |
| **PAHO** | 20,385 | 11,090 | 4,289 | 35,764 |
|  |  |  |  | *0.7%* |
| **SEARO** | 2,279,559 | 1,240,163 | 479,620 | 3,999,341 |
|  |  |  |  | *72.9%* |
| **WPRO** | 170,654 | 92,842 | 35,906 | 299,402 |
|  |  |  |  | *5.5%* |
| **Total** | **3,128,082** | **1,701,790** | **658,149** | **5,488,021** |
|  | *57.0%* | *31.0%* | *12.0%* |  |
| *AFRO: WHO African Region, EMRO: WHO Eastern Mediterranean Region, PAHO: WHO Pan-American Region, SEARO: WHO South-East Asian Region, WPRO: WHO Western Pacific Region, USDoS: United States Department of State, ILOSTAT: International Labour Organization Statistics. Costs are in 2016 US$ prices.* | | | | |

| **Supporting Table S6: Days lost to disease sequelae** | | | | |
| --- | --- | --- | --- | --- |
| **Region** | **Hydrocoele (thousands)** | **Lymphoedema (thousands)** | **ADL episodes (thousands)** | **Total days lost (thousands)** |
| **AFRO** | 460,938 | 250,767 | 96,982 | 808,687 |
|  |  |  |  | *32.4%* |
| **EMRO** | 26,343 | 14,332 | 5,543 | 46,218 |
|  |  |  |  | *1.9%* |
| **PAHO** | 9,807 | 5,335 | 2,063 | 17,206 |
|  |  |  |  | *0.7%* |
| **SEARO** | 873,424 | 475,174 | 183,769 | 1,532,366 |
|  |  |  |  | *61.4%* |
| **WPRO** | 52,006 | 28,293 | 10,942 | 91,241 |
|  |  |  |  | *3.7%* |
| **Total** | **1,422,518** | **773,901** | **299,298** | **2,495,717** |
|  | *57.0%* | *31.0%* | *12.0%* |  |
| *AFRO: WHO African Region, EMRO: WHO Eastern Mediterranean Region, PAHO: WHO Pan-American Region, SEARO: WHO South-East Asian Region, WPRO: WHO Western Pacific Region, USDoS: United States Department of State, ILOSTAT: International Labour Organization Statistics.* | | | | |

| Supporting Table S7: Breakdown of direct costs by disease sequelae | | | | | | | | | | | | |
| --- | --- | --- | --- | --- | --- | --- | --- | --- | --- | --- | --- | --- |
| **Region** | **Direct healthcare costs  (US$ thousands)** | | | | | **Patient medical expenses  (US$ thousands)** | | | | | **Total direct costs  (US$ thousands)** | |
|  | ADL episodes | Hydrocoele | | Lymph-oedema | | ADL episodes | | Hydrocoele | Lymph-oedema | |  |  |
| **AFRO** | 64,897 | 11,346 | | 11,913 | | 23,768 | | 7,893 | 4,736 | | 124,551 | |
|  |  |  |  |  |  |  |  |  |  |  | *44.9%* | |
| **EMRO** | 194 | 34 | | 36 | | 2,188 | | 819 | 491 | | 3,761 | |
|  |  |  |  |  |  |  |  |  |  |  | *1.4%* | |
| **PAHO** | 137 | 24 | | 25 | | 2,090 | | 966 | 580 | | 3,822 | |
|  |  |  |  |  |  |  |  |  |  |  | *1.4%* | |
| **SEARO** | 36,221 | 13,923 | | 13,656 | | 53,699 | | 12,521 | 7,513 | | 137,533 | |
|  |  |  |  |  |  |  |  |  |  |  | *49.6%* | |
| **WPRO** | 1,808 | 316 | | 332 | | 3,356 | | 1,036 | 622 | | 7,470 | |
|  |  |  |  |  |  |  |  |  |  |  | *2.7%* | |
| **Total** | **103,256** | **25,642** | | **25,961** | | **85,101** | | **23,235** | **13,941** | | **277,137** | |
|  | *37.3%* | *9.3%* | | *9.4%* | | *30.7%* | | *8.4%* | *5.0%* | |  |  |
| *AFRO: WHO African Region, EMRO: WHO Eastern Mediterranean Region, PAHO: WHO Pan-American Region, SEARO: WHO South-East Asian Region, WPRO: WHO Western Pacific Region, USDoS: United States Department of State, ILOSTAT: International Labour Organization Statistics. Costs are in 2016 US$ prices.* | | | | | | | | | | | | |
| Supporting Table S8: Total economic burden compared by wage source | | | | | | | | | | | |  |
| **Wage source** | | | **Direct healthcare costs  (US$ millions)** | | **Patient medical expenses  (US$ millions)** | | **Productivity costs  (US$ millions)** | | | **Total economic burden (US$ millions)** | |  |
| GDP per capita of lowest Quintile | | | 154.86 | | 122.28 | | 5,488.02 | | | **5,765.16** | |  |
| USDoS minimum wage | | | 154.86 | | 122.28 | | 6,932.57 | | | **7,209.71** | |  |
| ILOSTAT minimum wage | | | 154.86 | | 122.28 | | 6,095.00 | | | **6,372.14** | |  |
| *USDoS: United States Department of State, ILOSTAT: International Labour Organization Statistics. Costs are in 2016 US$ prices.* | | | | | | | | | | | |  |

| Supporting Table S9: Average economic burden per chronic morbidity | | | | | | | |
| --- | --- | --- | --- | --- | --- | --- | --- |
| **Region** | **Hydrocoele** | | | **Lymphoedema** | | | **Average economic burden per chronic case** |
|  | Direct healthcare costs | Patient medical expenses | Productivity costs | Direct healthcare costs | Patient medical expenses | Productivity costs |  |
| **AFRO** | US$1.31 | US$0.91 | US$67.16 | US$2.29 | US$0.91 | US$36.54 | **US$67.40** |
|  |  |  |  |  |  |  |  |
| **EMRO** | US$0.07 | US$1.65 | US$149.36 | US$0.12 | US$1.65 | US$81.26 | **US$145.87** |
|  |  |  |  |  |  |  |  |
| **PAHO** | US$0.13 | US$5.23 | US$110.31 | US$0.23 | US$5.23 | US$60.01 | **US$111.85** |
|  |  |  |  |  |  |  |  |
| **SEARO** | US$0.85 | US$0.76 | US$138.51 | US$1.38 | US$0.76 | US$75.35 | **US$135.47** |
|  |  |  |  |  |  |  |  |
| **WPRO** | US$0.32 | US$1.06 | US$174.15 | US$0.56 | US$1.06 | US$94.74 | **US$169.53** |
|  |  |  |  |  |  |  |  |
| **Total** | **US$0.96** | **US$0.87** | **US$116.70** | **US$1.61** | **US$0.87** | **US$105.81** | **US$114.69** |
| *AFRO: WHO African Region, EMRO: WHO Eastern Mediterranean Region, PAHO: WHO Pan-American Region, SEARO: WHO South-East Asian Region, WPRO: WHO Western Pacific Region. Costs are in 2016 US$ prices.* | | | | | | | |

## Sensitivity Analyses

Univariate sensitivity analyses were performed on the health burden and economic burden (Table 1; Supporting Table S3; Supporting Figure S2). Ranges for the values were based on those in previous analyses or found within the literature. Parameters were varied individually within each disease sequelae, except those relating to treatment-seeking behaviour and the disability weights, where the respective parameters for all disease sequelae were minimised/maximised.

**Supporting Figure S2. Tornado plot illustrating the impact of univariate sensitivity analysis on estimated pre-control total health (A) and economic burden of LF (B)**. *All parameters used for the sensitivity analysis are included in Table 1 and Supporting Table S3.*

**References**

1. Chu, B.K., et al., *The economic benefits resulting from the first 8 years of the Global Programme to Eliminate Lymphatic Filariasis (2000-2007).* PLoS Negl Trop Dis, 2010. **4**(6): p. e708.

2. Turner, H.C., Bettis, A.A., Chu, B.K., McFarland, D.A., Hooper, P.J., Ottesen, E.A. and Bradley, M.H. , *The Health and Economic Benefits of the Global Programme to Eliminate Lymphatic Filariasis (2000–2014).* Infect Dis Poverty, 2016. **5**(1): p. 54.

3. Ramaiah, K.D., et al., *Epidemiology of acute filarial episodes caused by Wuchereria bancrofti infection in two rural villages in Tamil, Nadu, south India.* Trans R Soc Trop Med Hyg, 1996. **90**(6): p. 639-43.

4. Babu, B.V., A.N. Nayak, and K. Dhal, *Epidemiology of episodic adenolymphangitis: a longitudinal prospective surveillance among a rural community endemic for bancroftian filariasis in coastal Orissa, India.* BMC Public Health, 2005. **5**: p. 50.

5. Gyapong, J.O., M. Gyapong, and S. Adjei, *The epidemiology of acute adenolymphangitis due to lymphatic filariasis in northern Ghana.* Am J Trop Med Hyg, 1996. **54**(6): p. 591-5.

6. Gasarasi, D.B., et al., *Acute adenolymphangitis due to bancroftian filariasis in Rufiji district, south east Tanzania.* Acta Trop, 2000. **75**(1): p. 19-28.

7. World Health Organization. *Cost effectiveness and strategic planning (WHO-CHOICE): Health service delivery costs*. Available from: <http://www.who.int/choice/cost-effectiveness/inputs/health_service/en/>.

8. Addiss, D.G. and M.A. Brady, *Morbidity management in the Global Programme to Eliminate Lymphatic Filariasis: a review of the scientific literature.* Filaria J, 2007. **6**: p. 2.

9. *Informal consultation on preventing disability from lymphatic filariasis, WHO, Geneva, August 2006.* Wkly Epidemiol Rec, 2006. **81**(40): p. 373-83.

10. Behera, M., S. Das, and J.K. Panda. *Current Management: Filariasis*. 2017; Available from: <http://webcache.googleusercontent.com/search?q=cache:http://www.apiindia.org/pdf/medicine_update_2017/mu_017.pdf>.

11. Management Sciences for Health. *The International Medical Products Price Guide,*. Available from: <http://mshpriceguide.org/en/home/>.

12. Gyapong, M., et al., *The burden of hydrocele on men in Northern Ghana.* Acta Trop, 2000. **77**(3): p. 287-94.

13. Babu, B.V., et al., *The economic loss due to treatment costs and work loss to individuals with chronic lymphatic filariasis in rural communities of Orissa, India.* Acta Trop, 2002. **82**(1): p. 31-8.

14. Nanda, B. and K. Krishnamoorthy, *Treatment seeking behaviour and costs due to acute and chronic forms of lymphatic filariasis in urban areas in south India.* Trop Med Int Health, 2003. **8**(1): p. 56-9.

15. Krishnamoorthy, K., *Estimated costs of acute adenolymphangitis to patients with chronic manifestations of bancroftian filariasis in India.* Indian J Public Health, 1999. **43**(2): p. 58-63.

16. World Bank. *World Development Indicators*. Available from: <http://data.worldbank.org/data-catalog/world-development-indicators>.

17. United States Department of State Country Reports on Human Rights Practices. Available from: <http://www.state.gov/j/drl/rls/hrrpt/>.

18. International Labour Organization’s Minimum Wages Database. Available from: <http://www.ilo.org/ilostat>.
